# Medication Errors as a Marker of Resident Competency

**DOI:** 10.7759/cureus.67829

**Published:** 2024-08-26

**Authors:** Oswaldo E Subillaga, Kenneth Lynch, Ashlie Haas-Rodriguez, David Harrington, Thomas Miner

**Affiliations:** 1 Department of Surgery, The Warren Alpert Medical School of Brown University, Brown University, Providence, USA

**Keywords:** general surgery, entrustable professional activities, acgme core competencies, internship and residency, medication errors, competency-based medical education (cbme)

## Abstract

Introduction

Educators continue to evaluate ways to assess resident performance in conjunction with the Accreditation Council for Graduate Medical Education (ACGME) general surgery milestones. We investigated whether the rate of medication errors could reflect general surgery resident competency. We hypothesized that the identification of increased medication errors made by general surgery residents could be a potential screening tool to identify residents who are academically at risk prior to their formal biannual milestone evaluation by the clinical competency committee.

Methods

This is a retrospective cohort study comparing rates of medication ordering errors against ACGME core competency scores over four years in a general surgery residency program at an academic, university-affiliated, level 1 trauma center in the Northeastern United States.

Results

We identified 95 general surgery residents who inputted 1,164,663 medication orders during the four years studied. There were 1,214 (0.1%) errors identified. Of those, 1,146 (94.4%) were level 3 errors, and 68 (5.6%) were level 4 errors*.* This represents an error rate of 1.04 errors per 1,000 medication orders. There was a statistically significant decrease in the error rate as the post-graduate year (PGY) level increased (p=0.005). However, there was no correlation between the error rate and individual ACGME milestone competency scores by PGY level.

Conclusions

We explored whether medication errors may be an early measurement of worsening resident performance as demonstrated by a decrease in ACGME core competency scores. However, the rate of errors did not correlate consistently with these measures. This may underscore that medication errors measure an aspect of resident performance that we do not capture with our current assessments.

## Introduction

The education of surgical residents has undergone significant changes over the past two decades, shifting in focus to competency-based education and an emphasis on patient safety and error-free practice. This effort was best highlighted in the 2001 American Board of Medical Specialties (ABMS) and Accreditation Council for Graduate Medical Education (ACGME) joint report on surgical competencies and the criteria needed for maintenance of certification [1]. The impetus for this initiative was in part driven by the Institute of Medicine's original publication To Err Is Human, which called for immediate action to reduce the number of deaths in the United States attributed to medical error [2]. During that time, the ACGME implemented its outcomes project delineating the six specific core competencies of medical training against which all residents would be assessed [3]. Since the rollout of the 2013 ACGME New Accreditation Program, the six core competencies have been expanded to 18 milestones for general surgery, ranging from knowledge, skills, attitudes, and other attributes that can be mapped to one of these core competencies [1,4,5].

The milestones are structured around a framework that ranges from less to more advanced, targeting a resident's performance as they move from intern year through their final year of training [1]. Surgical faculty and program leadership independently evaluate these milestones every six months. In the current training landscape, program directors continue to evaluate novel ways to assess resident performance in conjunction with these milestones, especially to identify residents who may be trailing behind their peers. The occurrence of preventable medication errors may be one way to identify at-risk surgical residents to institute an early corrective action plan [3]. Preventable medication errors have been reported at an average rate of 450,000 each year in the United States and with an estimated cost of over $2 billion [6,2]. These have been reported to represent 6.7-7.5% of total medication orders placed [7,8]. There are five phases within the broad definition of medication errors: prescription, transcription, dispensing, administering, and documentation [9]. Within the prescription or ordering phase, various error rates ranging from 4.2% to 6.7% have been reported [10-12]. Up to 10% higher rates of errors have been reported in teaching hospitals in the United States [13].

The purpose of this study is to evaluate the rates of inpatient medication errors made during the ordering or prescribing phase, hereinafter "medication errors," that occurred over four years in a general surgery residency program at an academic, university-affiliated, level 1 trauma center in the Northeastern United States. Our hypothesis is that medication errors can be used as a surrogate marker of competency for residents in a general surgery training program. Thus, we asked the following questions: Does a decrease in medication errors reflect increasing competency in the domain of medication orders? Does the rate of medication errors correlate with any individual general surgery core competency scores?

## Materials and methods

This study was approved by the Lifespan Research Protection Office (approval number: 406921). Following approval, the databases maintained by both the pharmacy department and the general surgery residency program were used to identify participants. Our study included every general surgery resident, both categorical and preliminary, who trained in our institution between academic years 2017 and 2021. We excluded orders placed by residents providing clinical coverage during their dedicated research years, hereinafter "research residents," as there was no milestone data available during their research time.

Total medication orders placed by general surgery residents between January 2017 and September 2020 were included in the analysis. Once errors are flagged and intervened upon, our institution's pharmacy department assigns error levels based on increasing potential severity index on a scale from 1 to 4. Level 1 errors represent no risk of harm to the patient (i.e., medication was ordered in tablet form instead of the capsule form), while level 2 errors represent a minimal risk of harm to the patient (i.e., 100 mg sertraline ordered for a patient whose home dose is 25 mg). Level 3 errors represent a high potential risk of harm to the patient and require increased monitoring (i.e., 20 units of insulin lispro ordered at bedtime, instead of insulin glargine as was intended), while level 4 errors represent a definite risk of harm to the patient (i.e., medication ordered for a patient with a documented anaphylactic allergy to that medication). Only level 3 and 4 errors are reported to the respective academic departments by the pharmacy. These results were exported and analyzed using descriptive statistics.

Next, resident general surgery milestone scores were evaluated. These scores were aggregated and averaged by their respective ACGME core competency for ease of both data collection and analysis. The average scores were thus recorded as follows: interpersonal and communication skills (IPC), professionalism (PROF), medical knowledge (MK), patient care (PC), systems-based practice (SBP), and practice-based learning and improvement (PBLI).

The primary outcome of this study was to evaluate whether a decreasing rate of medication ordering/prescribing errors, hereinafter "error rate," reflects increasing resident competency. Another outcome includes the potential correlation in the error rate in relation to ACGME core competency scores. Thus, the error rate was compared against the resident core competency scores to assess for correlation using Pearson's coefficient.

## Results

We identified data for 95 unique general surgery residents during the four years studied: post-graduate year 1 (PGY1) (N=63; 66.3%), PGY2 (N=45; 47.4%), PGY3 (N=35; 36.8%), PGY4 (N=33; 34.7%), and PGY5 (N=33; 34.7%). Our institution hosts seven preliminary PGY1 positions and two preliminary PGY2 positions, which explains the larger number of junior residents. Recorded medical orders were 1,223,220, of which 58,557 (4.8%) were placed by research residents and thus excluded. In total, 1,297 (0.1%) errors were recorded, of which research residents placed 83 (6.4%) (level 3: N=76, level 4: N=7) and were thus excluded. Our analysis was based on 1,164,663 orders placed, of which 1,214 (0.1%) contained errors. Of those errors, level 3 errors (N=1,146; 94.4%) and level 4 errors (N=68; 5.56%) were identified. This represents an error rate of 1.04 errors per 1,000 medication orders. Table 1 displays the data collected.

**Table 1:** Medication errors by PGY level PGY: post-graduate year

Errors	PGY1 (N=55)	PGY2 (N=40)	PGY3 (N=35)	PGY4 (N=33)	PGY5 (N=33)	P-value
Medication orders	325,164 (27.9%)	291,803 (25.1%)	242,974 (20.9%)	164,129 (14.1%)	140,593 (12.1%)	0.000023
Total medication errors (N=1214)	363 (29.9%)	358 (29.5%)	229 (18.9%)	143 (11.8%)	121 (10.0%)	0.000016
Level 3 errors (N=1146)	350 (30.5%)	337 (29.4%)	211 (18.4%)	135 (11.8%)	113 (9.8%)	0.000044
Level 4 errors (N=68)	13 (19.1%)	21 (30.9%)	18 (26.5%)	8 (11.8%)	8 (11.8%)	0.28
Average number of medication errors per resident	6.60	8.95	6.54	4.33	3.67	0.000016
Average error rate	1.26	1.21	0.94	0.89	0.79	0.0051

While the number of orders placed decreased with increasing seniority, PGY4 and PGY5 residents still accounted for a combined 26.2% of the total number of orders recorded. There was a statistically significant decrease in the error rate as the PGY level increased (p=0.005). This demonstrated a strong linear correlation with an R2 value of 0.9248.

The average core competency scores demonstrated a statistically significant increase as the PGY level increased (Table 2). These were inversely related to the decrease in error rate. However, the Pearson correlation analysis showed either no correlation or a very weak correlation between the error rate and the average score for each individual core competency by PGY level (Table 3).

**Table 2:** Average competency scores per PGY level IPC: interpersonal and communication skills; PROF: professionalism; MK: medical knowledge; PC: patient care; SBP: systems-based practice; PBLI: practice-based learning and improvement; PGY: post-graduate year

Core competency	PGY1 (N=60)	PGY2 (N=44)	PGY3 (N=35)	PGY4 (N=34)	PGY5 (N=34)	P-value
IPC	0.86	1.85	2.95	3.95	5.05	<0.05
PROF	0.95	1.93	3.04	4.08	5.09	<0.05
MK	0.89	1.68	2.73	3.48	4.75	<0.05
PC	0.81	1.63	2.69	3.89	5.22	<0.05
SBP	0.84	1.70	2.87	3.80	4.99	<0.05
PBLI	0.99	1.82	2.74	4.01	4.93	<0.05

**Table 3:** Pearson correlation between error rate and ACGME core competency scores, by PGY level Correlation by Pearson's coefficient: no correlation: 0, very weak: 0.01-0.19, weak: 0.20-0.39, moderate: 0.40-0.59, strong: 0.60-0.79, very strong: 0.80-1.00 IPC: interpersonal and communication skills; PROF: professionalism; MK: medical knowledge; PC: patient care; SBP: systems-based practice; PBLI: practice-based learning and improvement; ACGME: Accreditation Council for Graduate Medical Education; PGY: post-graduate year

Core competency	PGY1 (N=52)	PGY2 (N=39)	PGY3 (N=35)	PGY4 (N=33)	PGY5 (N=33)
IPC	-0.16	0.03	0.04	-0.15	-0.10
PROF	-0.07	0.00	-0.22	-0.17	-0.22
MK	-0.15	0.00	-0.31	-0.25	-0.23
PC	0.12	0.05	0.08	-0.34	-0.24
SBP	-0.12	-0.08	-0.25	-0.08	-0.18
PBLI	0.14	0.12	0.12	-0.31	0.03

## Discussion

Over the past two decades, the ACGME and the American Board of Surgery (ABS) have implemented directives to improve competency-based assessments in training programs. For competency-based medical education to benefit residents, they must receive timely, actionable, and high-quality feedback to help guide their development and achieve competency in a respective domain [14,15]. Our results showed a significant decrease in the rate of medication errors as PGY increased. While this is an encouraging finding, are we able to say that our residents are competent in the domain of medication order entry upon graduation?

To better elucidate an answer to this question, one can turn to the Dreyfus developmental model which posits that learners pass through five levels of skill acquisition through formal education and practice: novice, advanced beginner, competent, proficient, and expert [16]. Since its initial publication, the Dreyfus model has been adapted for use in virtually all disciplines but has had substantial adaptation within the disciplines of nursing and medicine [17,18]. This model has particular significance to competency-based medical education, surgical training, and the findings of this study.

An assumption exists that residents become more competent as they progress through training. According to the Dreyfus model, "to avoid mistakes, the competent performer seeks rules and reasoning procedures to decide which plan or perspective to adopt" [19]. Our results provide statistically significant data that support the assumption that surgery residents make linear progression in their milestone competency scores and in the domain of medication order entry across five years of training based on our current assessment and evaluation techniques.

In this study, we further hypothesized that the identification of increased medication errors made by general surgery residents could be a potential screening tool to identify residents who are at risk or failing at their position prior to their formal biannual ACGME milestone evaluation by the clinical competency committee. This would allow for earlier intervention and possible remediation before falling further behind their peers in training. However, our analysis found no correlation between the error rate and any of the averaged core competency scores. While some core competency scores displayed a weak correlation with the error rate (i.e., MK during four out of the five training years), there was no consistent pattern (Table 3).

The lack of consistent correlation between error rates and core competency scores raises the question of whether error rates are measuring an aspect of resident performance that is not being evaluated with the assessment tools currently in place. We might consider whether the current milestones that cover knowledge of pathophysiology, non-surgical treatment, and reporting of errors could benefit from including these concrete and granular data to which our departments have easy access in the era of electronic medical records [4]. This question remains unsettled, however, and would benefit from future studies including a larger and more diverse pool of residents.

These results are limited as they represent only one academic institution. Additionally, error data was not available for a small number of residents as some were mistakenly assigned to different clinical departments in internal rosters. Additionally, the possibility exists that decreasing error rates are affected by familiarity with an institution's electronic medical record as residents progress in their training. Finally, while similar studies have reported on all errors, we only report on those categorized as higher risk (levels 3 and 4) and thus have a reduced sample of errors relative to the total number of orders [10].

## Conclusions

Decreasing rates of medication errors likely demonstrate increasing competency as residents progress through their training. However, it did not show a correlation with any ACGME core competency. Our results may be signaling an aspect of resident performance not currently being measured by the assessment tools available to general surgery residency programs. If we want to continue to advance competency-based medical education in surgical training, we need to continue seeking objective measures of resident performance to determine competency. Though more research is needed, medication errors provide granular, objective data that may be incorporated into surgery resident assessment and evaluation.
